# From COPD to cancer: indacaterol’s unexpected role in combating NSCLC

**DOI:** 10.3389/fphar.2025.1579126

**Published:** 2025-04-03

**Authors:** Chenghao Liu, Jiaqi Huang, Pengjie Cai, Min Jiang, Honglei Chen

**Affiliations:** ^1^ Department of Pathology, School of Basic Medical Sciences, Wuhan University, Wuhan, China; ^2^ Department of Pathology, Zhongnan Hospital of Wuhan University, Wuhan, China; ^3^ Department of Laboratory Medicine, Tinghu District Center for Disease Control and Prevention, Yancheng, China; ^4^ Karamay Central Hospital of Xinjiang, Xinjiang Key Laboratory of Clinical Genetic Testing and Biomedical Information, Karamay, China

**Keywords:** indacaterol, GLUT1, lung cancer, combination therapy, PD-L1 inhibitors

## Abstract

**Background:**

Non-small cell lung cancer (NSCLC) is one of the most prevalent and deadly malignancies worldwide. In previous studies, indacaterol, a drug used to manage chronic obstructive pulmonary disease, has shown antitumor activity. However, its role in the context of NSCLC remains underexplored. This study aimed to investigate indacaterol’s mechanisms and potential therapeutic effects in lung cancer treatment.

**Methods:**

Expression profiles and clinical information from the TCGA database were analyzed to explore the potential impact of the *SLC2A1* gene on the progression of NSCLC. The expression levels of the GLUT1 protein, encoded by the *SLC2A1* gene, and the MCT4 protein, encoded by the *SLC16A3* gene, were analyzed in both lung cancer and normal tissues. Techniques such as cellular thermal shift assay (CETSA), immunofluorescence, and Western blotting were employed to assess the interaction between indacaterol and GLUT1. Immunohistochemistry was used to study the expression of GLUT1 and MCT4 in human tissues. The effects of indacaterol on lung cancer cell lines were observed through wound healing and colony formation assays. Additionally, animal experiments combined with PD-L1 inhibitors were conducted to evaluate the antitumor effects of indacaterol *in vitro* and *in vivo*.

**Results:**

Analysis of TCGA data revealed that GLUT1 has a potential role in promoting NSCLC and may work in concert with MCT4. Indacaterol significantly inhibited the viability of NSCLC cells in a concentration-dependent manner. Molecular modeling and CETSA experiments further indicated that indacaterol may bind to GLUT1 and affect GLUT1 expression. Immunohistochemistry suggested that indacaterol also reduces the expression of MCT4, suggesting its potential to diminish the capacity of tumors to reprogram stromal metabolism. *In vitro* and *in vivo* experiments confirmed that the combination of indacaterol with PD-L1 inhibitors synergistically inhibited the proliferation and invasion of NSCLC cells.

**Conclusion:**

Indacaterol, a potential inhibitor of GLUT1, has significant antitumor effects on NSCLC. Moreover, the combination of indacaterol with immune checkpoint inhibitors may further enhanced the inhibitory effects of indacaterol on NSCLC cells. Our study provides scientific evidence supporting the clinical application of indacaterol as a novel therapeutic strategy for NSCLC treatment.

## 1 Introduction

Lung cancer is the most prevalent malignancy and the leading cause of cancer deaths worldwide (Bray et al., 2024). Current treatments—surgical resection, chemotherapy, radiotherapy, and targeted therapy—face limitations, including adverse effects, poor patient tolerance, and tumor recurrence (Li et al., 2023a). Non-small cell lung cancer (NSCLC) constitutes 80% of cases, where chemotherapy remains pivotal despite advances in targeted immunotherapies (Pirker, 2020). Its role in enhancing immunotherapy response and overcoming resistance underscores its clinical importance (Zouein et al., 2022), necessitating novel strategies to improve NSCLC outcomes.

Indacaterol, a long-acting β-agonist used in COPD management (Singh et al., 2022), demonstrates emerging antitumor potential. Studies reveal its ability to target SRSF6, suppressing progression and metastasis in colorectal (Wan et al., 2019) and breast cancers (Ayama-Canden et al., 2023). The COPD-lung cancer comorbidity, with COPD being an established risk factor (Forder et al., 2023), highlights the therapeutic relevance of investigating indacaterol’s antitumor mechanisms. Preliminary evidence suggests synergistic effects with gefitinib in epidermal growth factor receptor tyrosine kinase inhibitor-resistant cases (Li et al., 2017), though mechanistic details require further exploration.

Tumor cells exhibit enhanced glucose uptake/utilization compared to normal cells, with solute carrier (SLC) proteins overexpressed in colorectal, breast, and lung cancers (Alam et al., 2023; Cong et al., 2023). The *SLC2A1*-encoded GLUT1 critically regulates tumor proliferation and invasion, driving interest in GLUT1 inhibitors (Cao et al., 2021; Madunić et al., 2018). GLUT1, a pivotal glycolytic protein, is intricately associated with tumor metabolism in diverse immunotherapeutic contexts (Leone and Powell, 2020).

This study investigated indacaterol’s effects on lung cancer through GLUT1-mediated mechanisms. Integrating bioinformatics and experimental approaches, we evaluated its therapeutic potential and synergies with immune checkpoint inhibitors. These findings establish indacaterol’s translational framework for lung cancer and evidence for combination therapies.

## 2 Materials and methods

### 2.1 Data acquisition from TCGA

Pan-cancer TCGA expression data and clinical metadata (survival status/time, pathological stages) were obtained from Xena (https://xena.ucsc.edu/). Subsequent integration and analysis were performed using R 4.4.1.

### 2.2 Analysis of clinical features

Kaplan-Meier and Cox regression analyses (via *survival* and *survminer* packages) evaluated associations of *SLC2A1*/*SLC16A3* with overall (OS), disease-specific survival (DSS), disease-free (DFI), and progression-free (PFI) interval. Welch’s ANOVA assessed gene-clinical correlations, including pathological staging.

### 2.3 Immune analysis by TIMER 2.0

Immune cell infiltration was analyzed via TIMER2.0 (http://timer.cistrome.org/) with the TIMER, EPIC, QUANTISEQ, CIBERSORT, CIBERSORT-ABS, XCELL, MCPCOUNTER, and EPIC algorithms. All algorithms employed Spearman’s rank correlation coefficient, denoted by rho (ρ), for statistical analysis.

### 2.4 Functional analysis

In R 4.4.1, the GSVA package was used to process the ssGSEA data to calculate the activity status of the necrosis and apoptosis pathways in “c5.all.v7.4.symbols” from MSigDB. Lung adenocarcinoma (LUAD) gene set enrichment analysis (GSEA) utilized the BEST database (https://rookieutopia.hiplot.com.cn/app_direct/BEST/).

### 2.5 Gene correlation

In R 4.4.1, the Spearman method was used to analyze the associations between *SLC2A1* and its coexpressed genes in LUAD.

### 2.6 Cell culture

The H460, H1299, A549, and Lewis cell lines were purchased from Shanghai Zhong Qiao Xin Zhou Biotechnology Co., Ltd. The cells were washed with PBS, digested with trypsin, and neutralized with a 10% FBS medium after 2.5 min. The suspension was centrifuged, the supernatant was discarded, and the pellet was resuspended and replated in a fresh medium. PBS (MA0015), RPMI-1640 (MA0215), MEM (MA0217), and DMEM (MA0212) were obtained from Meilun Biotechnology (Dalian, China).

### 2.7 Wound healing assay

Cells were seeded in 6-well plates (5 × 10^5^ cells/well). After overnight incubation, scratches were made, and migration was assessed via image capture at 0, 24, and 48 h, with control and experimental wells receiving the complete medium and test drug, respectively.

### 2.8 Colony-formation assay

The cells were seeded at 100 cells/mL in six-well plates with drug-treated, serum-free media (experimental) or 10% FBS media (control). After 14 days, the colonies were fixed with 95% ethanol, stained with 0.1% crystal violet, and photographed.

### 2.9 Transwell assay

The Matrigel was thawed at 4°C and diluted 1:8 with DMEM, and 70 µL was added to the upper Transwell chamber. After 1–2 h of incubation at 37°C, the chambers were washed with PBS. The cells (5 × 10^4^/100 µL) were seeded and treated with drugs for 24 h. Chambers were then fixed and stained with 0.1% crystal violet, and the membrane was mounted for microscopy.

### 2.10 Fluorescence staining for necrosis detection

The cells were washed three times with staining buffer. Hoechst 33342 (5 μL) and PI (5 μL) were added to each well and mixed. The cells were stained at 4°C for 20–30 min, washed with PBS, and observed under a fluorescence microscope.

### 2.11 Flow cytometry for apoptosis detection

The cells were stained with Hoechst 33342 (1 μg/mL) and incubated at 37°C for 7–10 min. After centrifugation at 500–1,000 rpm for 5 min, the dye was removed. The cells were then stained with PI at 4°C for 15 min in the dark. The sample was filtered through a 400-mesh sieve. Flow cytometry data acquisition was performed on a CytoFLEX S instrument (Beckman Coulter, USA), followed by computational processing with FlowJo.

### 2.12 Schematic diagrams of binding modes

Molecular docking was performed via AutoDock 4.2 with the Lamarckian genetic algorithm. The structure of indacaterol was generated via the RDKit program, and partial charges were calculated via the AM1-BCC method. The structure of the GLUT1 protein was obtained from the RCSB PDB database (PDB code: 5EQG). Docking was carried out using a grid box large enough to encompass the binding site. The final GLUT1-indacaterol complex was obtained by aligning the structures via the align function in PyMOL.

### 2.13 Cellular thermal shift assay

Cells were incubated with varying concentrations of indacaterol to induce changes in GLUT1 thermal stability. The lysates were heated, and the remaining protein was quantified by Western blot analysis.

### 2.14 Animal experiments

Male C57BL/6 mice (6–8 weeks, 16–18 g; Wuhan Shoubei Biotech) were ethanol-sterilized and subcutaneously injected with 0.2 mL cell suspension (2 × 10^5^ cells). On day 10, 20 tumor-bearing mice were randomized into four groups: control (saline), indacaterol (0.0025 μg/g), PD-L1 inhibitor (2.500 μg/g), and combination treatment (200 μL daily). Body weight, stool, and tumor size were monitored biweekly. On day 14, mice were euthanized (cervical dislocation), and tumors/organs were harvested. The dosage regimen used in this study was derived from the Chinese patent application (Application No. 201910361231.8) titled “Use of Indacaterol Maleate in the Preparation of Anti-Tumor Pharmaceuticals”.

### 2.15 Mouse serum ELISA

Antibodies (0.1 mL) were incubated overnight at 4°C with samples. After washing, serum samples (0.1 mL) were incubated at 37°C for 1 h. Secondary antibodies were added, and incubation continued for 0.5–1 h at 37°C. TMB substrate was added, and OD values were measured at 450 nm.

### 2.16 HE staining

Tissue sections were deparaffinized, stained with hematoxylin for 2 min, and rinsed with water. Eosin staining was applied for 10–15 s, followed by dehydration in absolute ethanol. After drying, slides were mounted and observed under a microscope.

### 2.17 Selection of patients

This study analyzed 54 formalin-fixed, paraffin-embedded tissue samples from primary lung cancer patients without prior radiotherapy or chemotherapy. Clinical data were recorded, and two pathologists independently reviewed the samples twice. The study was approved by the Ethics Committee of Wuhan University School of Medicine, with consent obtained from participants or their representatives.

### 2.18 Immunohistochemical staining

Immunohistochemical staining assessed GLUT1 and MCT4 expression. After dewaxing, hydration, and antigen retrieval with citrate buffer (pH 6.0), sections were incubated with GLUT1 (1:200, HUABIO) and MCT4 (1:100, Santa Cruz) antibodies for 1.5 h at 37°C. Horseradish peroxidase and 3,3′-diaminobenzidine were applied, followed by hematoxylin counterstaining. PBS was used as a negative control.

### 2.19 Double immunofluorescent staining

Double immunofluorescent staining was performed using the Opal 7-Color IHC Kit (Akoya). GLUT1 (1:400, HUABIO) and MCT4 (1:200, Santa Cruz) were visualized via tyramide signal amplification. Stained slides were scanned and processed with InForm 2.6 software.

### 2.20 Statistical analysis

Statistical analysis was performed using GraphPad Prism 9.5. An unpaired Student’s t-test was used for two-group comparisons with normal distribution, and the Mann‒Whitney U test for nonnormal distributions. Comparisons among the three groups were analyzed using one-way ANOVA. Relationships between MCT4, GLUT1, and clinicopathological parameters were analyzed via the χ^2^ test.

## 3 Results

### 3.1 Expression patterns of the *SLC2A1* gene across pancancer types

**FIGURE 1:**
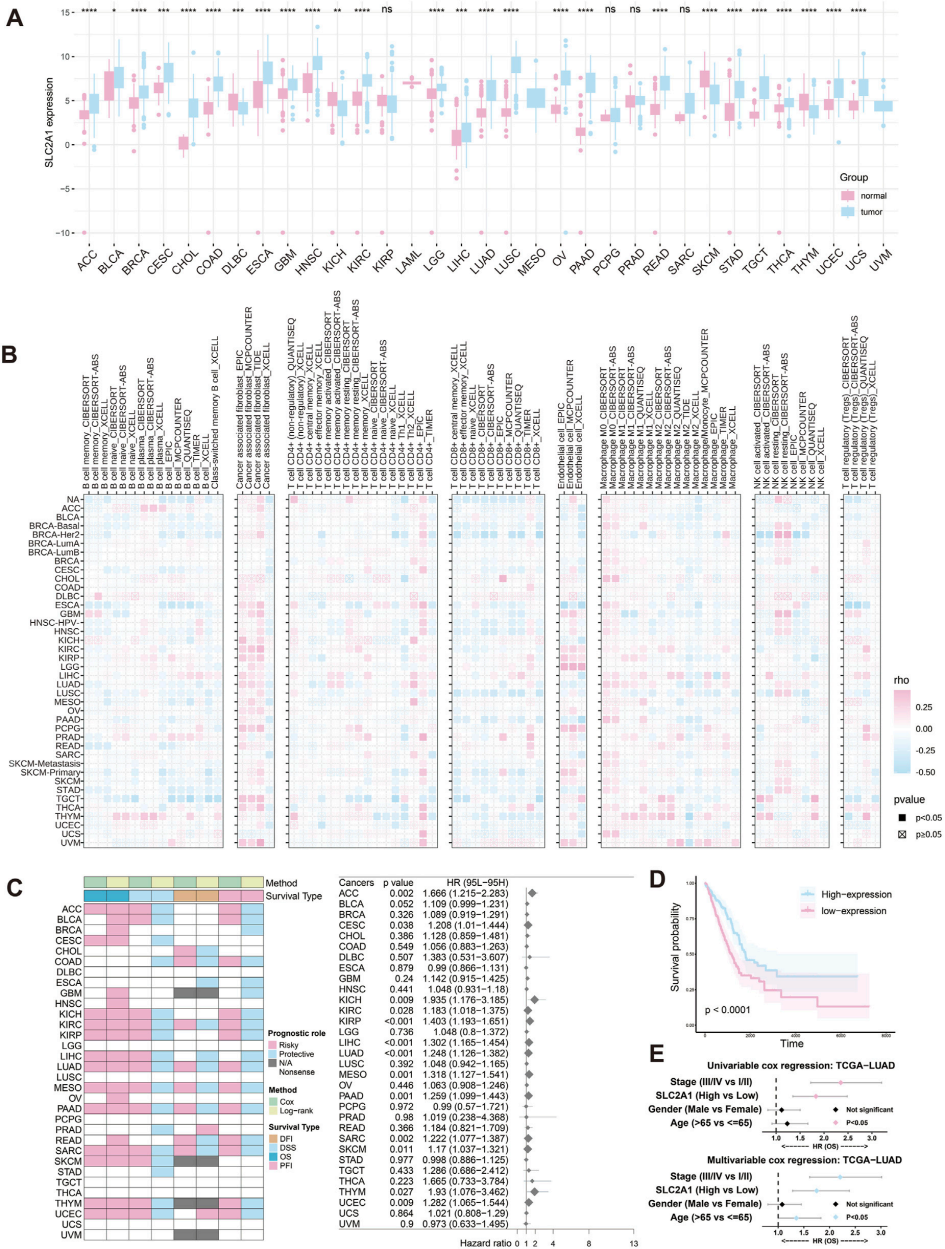
*SLC2A1* expression in pancancer and LUAD. **(A)** Expression levels of *SLC2A1* in tumor and normal tissues across various cancers. **(B)** Correlation of *SLC2A1* expression with immune cell infiltration across cancers. **(C)** Association of *SLC2A1* expression with patient survival and relative risk across cancers. **(D)** Kaplan‒Meier survival curves of the high and low *SLC2A1* expression groups in LUAD. **(E)** Univariate and multivariate Cox regression analyses of *SLC2A1* and clinical features in patients with LUAD. (*p < 0.05, **p < 0.01, ***p < 0.005, ****p < 0.001).

Analysis of the TCGA database revealed significant differences in *SLC2A1* (GLUT1) expression between tumor and normal tissues across various cancers. *SLC2A1* was upregulated in ACC, BRCA, CHOL, COAD, LUAD, and LUSC but downregulated in DLBC, SKCM, and THYM (Figure 1A).

The tumor microenvironment (TME) is the complex cellular environment surrounding tumor cells, composed of tumor and nontumor components that influence tumor progression and treatment response (Gargalionis et al., 2024). As a result, targeting the TME has become a key focus of drug development (Xiao and Yu, 2021). To explore the relationship between *SLC2A1* and the TME, we used various algorithms (Figure 1B). The analysis showed that *SLC2A1* is significantly associated with cancer-associated fibroblasts (CAFs) in several cancers, including ESCA, KIRC, LUAD, and TGCT. *SLC2A1* also correlates with immune suppression by affecting CD8^+^ T cell numbers, especially in LUSC and BRCA-Her, where a negative correlation was observed. Furthermore, *SLC2A1* negatively correlated with activated NK cells and positively with resting NK cells, suggesting its role in immune evasion. Based on these findings, we hypothesize that *SLC2A1* may remodel the TME, suppress immune responses, and promote tumor progression.

To investigate the impact of *SLC2A1* on cancer patient survival, we analyzed its correlation with OS, DSS, DFI, and PFI across cancer types (Figure 1C). High *SLC2A1* expression was significantly associated with decreased OS in ACC, KICH, KIRC, and LUAD. The relationships with DSS, DFI, and PFI varied based on the analytical methods used. Univariate analysis confirmed *SLC2A1* as a risk factor in several cancers, with hazard ratios of 1.666 for ACC, 1.935 for KICH, and 1.248 for LUAD.

In summary, *SLC2A1* is linked to poor prognosis in various cancers, especially LUAD, where it may serve as a prognostic biomarker. Using the median expression level as a threshold, we plotted survival curves from LUAD data in the TCGA database. The results showed that patients with high *SLC2A1* expression had significantly lower survival rates than those with low expression (p < 0.0001) (Figure 1D). Both univariate and multivariate analyses identified *SLC2A1* as an independent risk factor (Figure 1E). Given its critical role in LUAD, we further explored the impact of the *SLC2A1* gene and its encoded protein in lung cancer.

### 3.2 Functional analysis and clinical relevance of *SLC2A1* in LUAD

**FIGURE 2:**
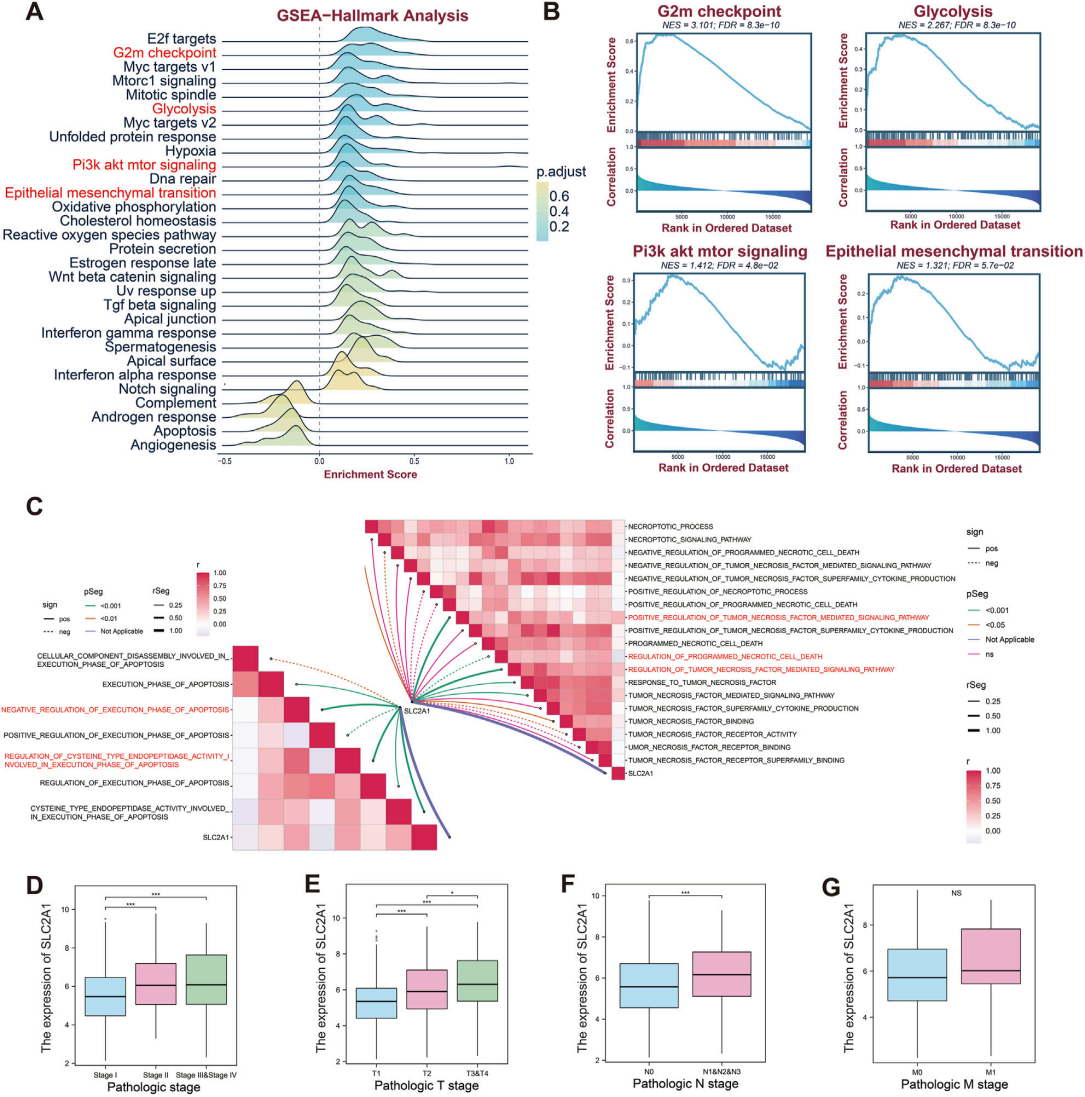
Relationships among *SLC2A1*, functions, and clinical staging in LUAD. **(A)** GSEA enrichment scores of *SLC2A1*-related hallmark pathways in LUAD. **(B)** GSEA of *SLC2A1* in the G2M checkpoint, glycolysis, PI3K/AKT/mTOR signaling, and epithelial-mesenchymal transition pathways in LUAD. **(C)** Correlations of *SLC2A1* with necrosis and apoptosis pathways in LUAD. *SLC2A1* expression in LUAD patients according to the pathological stage **(D)**, T stage **(E)**, N stage **(F)**, and M stage **(G)**. (*p < 0.05, **p < 0.01, ***p < 0.005).

To explore the potential role of *SLC2A1* in NSCLC, we performed gene set enrichment analysis (GSEA) using LUAD data from the TCGA database. The results indicated that *SLC2A1* may be linked to key cancer hallmarks, including the G2M checkpoint, glycolysis, the PI3K-AKT-mTOR pathway, and epithelial-mesenchymal transition (EMT) (Figure 2A). These hallmarks were further visualized (Figure 2B). We propose that *SLC2A1* may influence tumor growth and metastasis by modulating the G2M checkpoint and EMT while also regulating tumor metabolism through glycolysis and PI3K-AKT-mTOR signaling, enhancing tumor cell adaptability in hypoxic environments.

We then applied gene set variation analysis (GSVA) to gene sets related to apoptosis and necroptosis pathways. The GSVA results showed that *SLC2A1* is linked to various apoptotic pathways and negatively regulates apoptosis execution. Our analysis also indicated that *SLC2A1* regulates caspase activity (Figure 2C). Additionally, *SLC2A1* may influence tumor necrosis factors and is negatively correlated with programmed necrotic cell death modulation (Figure 2C).

When analyzing the clinical relevance of *SLC2A1* in LUAD, we observed significant variations in its expression across tumor stages. *SLC2A1* expression was higher in stages 2, 3, and 4 than in stage 1, suggesting a link to poor tumor prognosis (Figure 2D). Additionally, *SLC2A1* expression correlated with tumor size (T) and lymph node metastasis (N), with higher levels associated with advanced T and N stages. However, no such correlation was observed for distant metastasis (M) (Figure 2D–G).

In summary, our findings highlight the potential role of *SLC2A1* in LUAD progression and suggest its viability as a prognostic biomarker.

### 3.3 Indacaterol inhibits lung cancer cell viability and upregulates GLUT1

**FIGURE 3:**
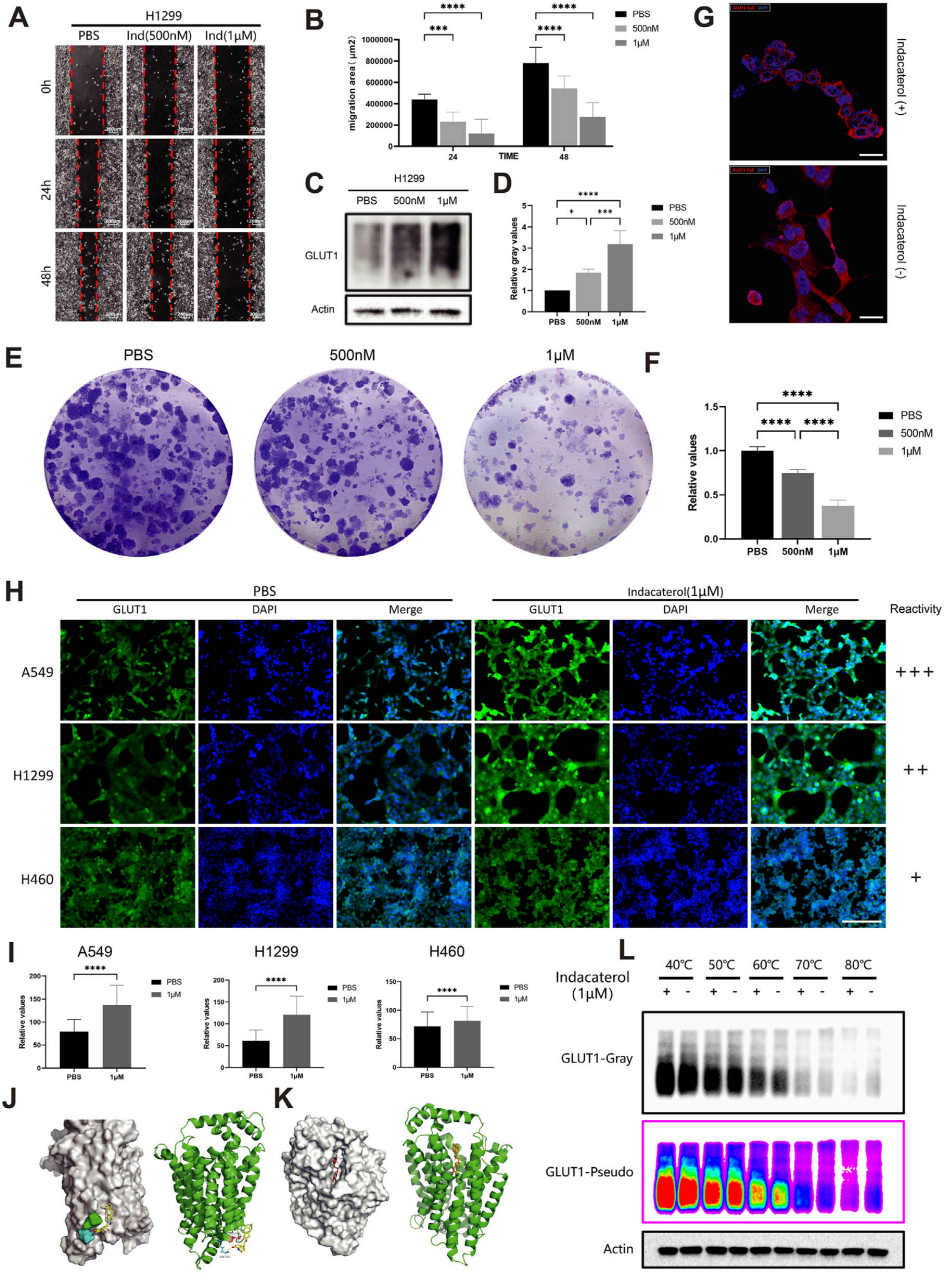
Indacaterol inhibits lung cancer cell lines through GLUT1. **(A)** Wound healing assay of H1299 cells treated with PBS, 500 nM, or 1 μM Indacaterol for 0, 24, or 48 h. **(B)** Quantitative analysis of the wound healing assay results. **(C)** Western blot of GLUT1 protein in H1299 cells treated with PBS, 500 nM, or 1 μM Indacaterol. **(D)** Quantitative analysis of the Western blot results. **(E)** Colony formation assay showing the effects of PBS, 500 nM, and 1 μM Indacaterol on A549 cells. **(F)** Quantitative analysis of the colony formation assay results. **(G)** Confocal microscopy images of GLUT1 expression and nuclear morphology: Indacaterol-treated cells (upper panel) versus untreated controls (lower panel). **(H)** Immunofluorescence staining of GLUT1 protein in A549, H1299, and H460 cells treated with PBS or 1 μM Indacaterol. Scale bars, 200 μm. **(I)** Quantitative analysis of the immunofluorescence results. **(J, K)** Schematic diagrams showing two binding modes of indacaterol and the GLUT1 protein. **(L)** Cellular thermal shift assay showing that Indacaterol binds to the GLUT1 protein. (*p < 0.05, **p < 0.01, ***p < 0.005, ****p < 0.001).

Indacaterol, commonly used in COPD management, has shown potential antitumor effects beyond its role in respiratory diseases (Wan et al., 2019). We assessed its impact on the migratory capacity of lung cancer cell lines, treating H1299 cells with 500 nM PBS, 500 nM indacaterol, or 1 μM indacaterol. Wound healing assays showed significant inhibition of migration with prolonged indacaterol exposure, with stronger effects at higher concentrations (Figure 3A, B). Western blot analysis demonstrated that GLUT1 protein expression increased with higher indacaterol concentrations (Figure 3C), a result confirmed by quantification (Figure 3D). To validate the results across different lung cancer cell lines, we conducted additional experiments. In A549 cells, colony formation assays showed that indacaterol significantly inhibited colony formation in a dose-dependent manner (Figure 3E, F).

Given the potential oncogenic role of *SLC2A1* (GLUT1) in LUAD, we speculate whether indacaterol can affect the biological behavior of lung cancer cells by regulating *SLC2A1* (GLUT1). Confocal microscopy revealed that A549 cells treated with Indacaterol exhibited higher peripheral GLUT1 signal intensity and more aberrant nuclear morphology (Figure 3G). Immunofluorescence analysis of A549, H1299, and H460 cells treated with PBS or indacaterol showed increased GLUT1 fluorescence intensity in A549 and H1299 cells, with a less pronounced effect in H460 cells (Figure 3H, I). This variability indicates that indacaterol-induced GLUT1 upregulation may be more prominent in certain lung cancer cell lines, suggesting these cells may be more sensitive to the drug.

Interestingly, during tumor cell death, most membrane proteins typically show a corresponding decrease in expression. However, in our study, we observed an abnormal increase in GLUT1 expression alongside the inhibition of lung cancer cell viability by indacaterol. This prompted further investigation.

Using the online predictive tool https://prediction.charite.de/subpages/target_prediction.php, we identified potential targets of indacaterol, which suggested that it might interact with the SLC family of proteins, with a predicted probability of 87.09% and model accuracy of 98.75%. Molecular docking simulations predicted a potentially stable binding mode between indacaterol and GLUT1 (Figure 3J, K). We next conducted a cellular thermal shift assay (CETSA), which revealed a notable shift in the thermal denaturation curve of GLUT1 between the drug-treated and untreated groups at 60°C, with increased nondegraded GLUT1 protein (Figure 3L). We performed grayscale validation of the CETSA results, which confirmed that indacaterol binds to the GLUT1 protein and induces a thermal shift in its melt curve (Supplementary Figure S1). We also examined other metabolism-related proteins in the H1299 cell line under different treatment conditions and found that indacaterol inhibited AKT phosphorylation while increasing β-arrestin expression (Supplementary Figures S2A, B). Analysis of LUAD samples revealed that GLUT1 expression was positively correlated with AKT1 (p = 5.8e-16, r = 0.26) and AKT2 (p = 1.3e-44, r = 0.43) and negatively correlated with ARRN1 (p = 1.1e-138, r = −0.69) and ARRB2 (p = 2.3e-35, r = −0.38) expression (Supplementary Figure S2C). AKT, a key component of the dysregulated PI3K pathway, regulates GLUT1 phosphorylation (Revathidevi and Munirajan, 2019; Fontana et al., 2024). β-arrestin, also involved in tumor progression, participates in metabolic processes, including glucose metabolism (Kim et al., 2023). These findings indicate that indacaterol binds to GLUT1 protein and upregulates its expression, and may also influence tumor development by regulating the metabolism of lung cancer cells, however, the main mechanism by which indacaterol inhibits tumors remains unclear.

### 3.4 Indacaterol inhibits GLUT1 co-expressed protein MCT4

**FIGURE 4:**
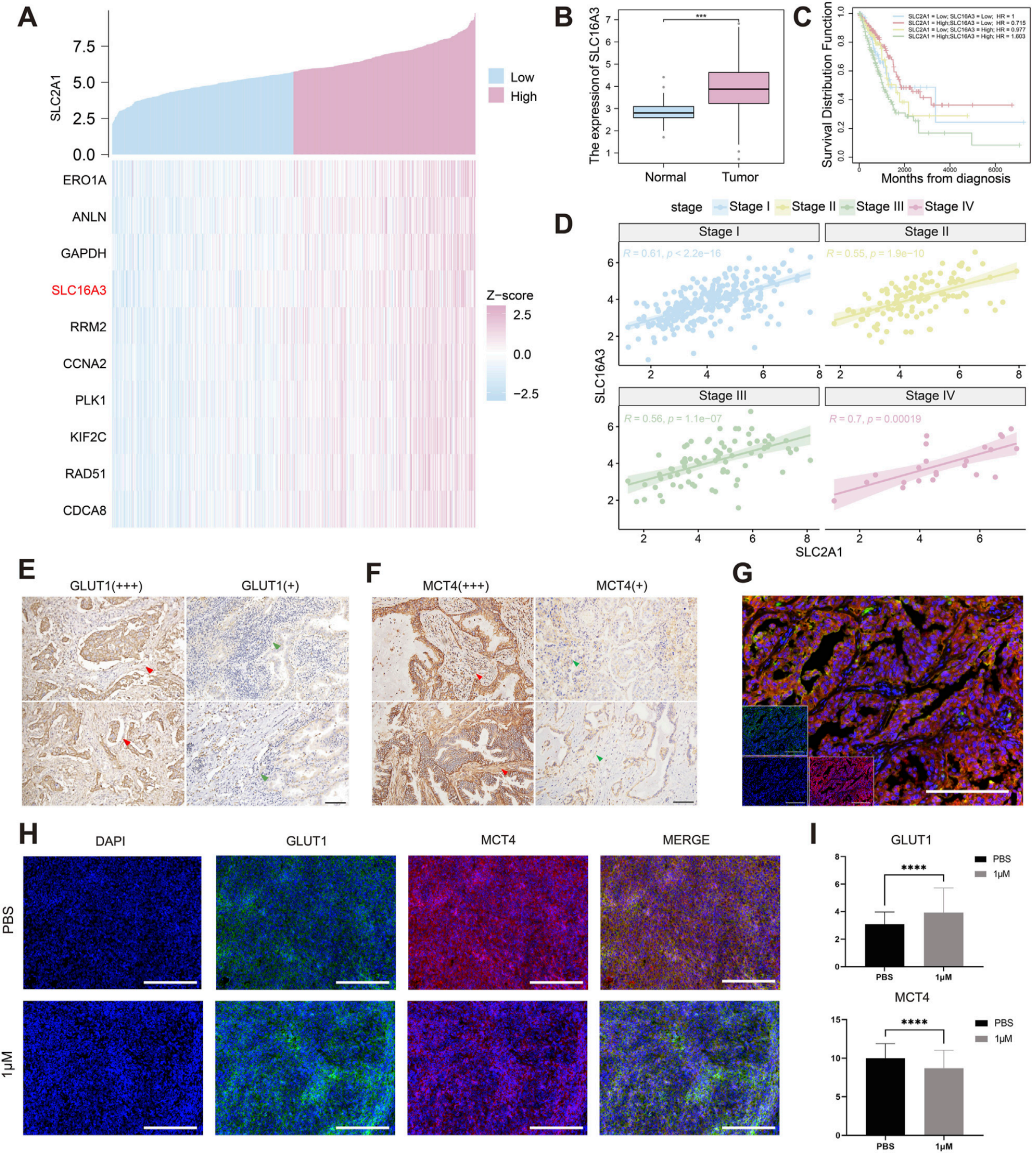
Indacaterol affects the expression of both GLUT1 and MCT4. **(A)** Coexpression analysis of *SLC2A1* in LUAD. **(B)** Expression levels of *SLC16A3* in tumor and normal tissues in LUAD. **(C)** Kaplan‒Meier curves of *SLC16A3* and *SLC2A1* in LUAD. **(D)** Pearson correlation analysis of *SLC16A3* and *SLC2A1* expression across different LUAD stages. **(E–F)** Immunohistochemistry results show high and low expression of GLUT1 and MCT4 proteins in human lung cancer tissues. Scale bars, 100 μm. **(G)** Multiplex immunofluorescence staining of GLUT1 and MCT4 proteins in human lung cancer tissues. Scale bars, 200 μm. **(H)** Immunofluorescence staining of GLUT1 and MCT4 proteins in the lung cancer tissues of the mice treated with PBS or 1 μM Indacaterol. Scale bars, 200 μm. **(I)** Quantitative analysis of the immunofluorescence results. (*p < 0.05, **p < 0.01, ***p < 0.005, ****p < 0.001).

We further explored how indacaterol affects lung cancer and its impact on GLUT1. We identified coexpressed genes associated with *SLC2A1* in LUAD and highlighted the top 10 genes with the highest correlation coefficients (Figure 4A). Notably, *SLC16A3*, a gene from the same family as *SLC2A1*, emerged as a key finding. In LUAD, *SLC2A1* expression was significantly positively correlated with *SLC16A3* expression (r = 0.6, p < 2.2e-6) (Supplementary Figure S3A).

*SLC16A3* encodes MCT4, a member of the solute carrier family 16, which transports lactate out of cells to prevent intracellular accumulation that would inhibit glycolysis, supporting the high glycolytic phenotype and invasiveness of tumor cells and maintaining an acidic TME (Singh et al., 2023). Inhibiting MCT4 enhances immune checkpoint inhibitor efficacy (Babl et al., 2023). The coexpression of GLUT1 and MCT4 promotes cancer progression in various cancers, such as ovarian cancer (Baczewska et al., 2022) and hepatocellular carcinoma (Granja et al., 2022). In LUAD, *SLC16A3* expression was higher than in normal tissue (Figure 4B), and patients with high *SLC16A3* expression had significantly lower survival rates (p < 0.0001) (Supplementary Figure S3B). Based on *SLC2A1* and *SLC16A3* expression, LUAD patients were categorized into four groups: *SLC2A1*high*SLC16A3*low, *SLC2A1*low*SLC16A3*high, *SLC2A1*high*SLC16A3*high, and *SLC2A1*low*SLC16A3*low. Survival analysis showed that patients with high expression of both genes had the poorest prognosis (Figure 4C). A strong correlation between *SLC2A1* and *SLC16A3* was found in all stages of LUAD, especially in stage IV (r = 0.7, p = 0.00019) (Figure 4D). These findings suggest that *SLC2A1* and *SLC16A3* may synergistically promote tumor progression and reduce survival in lung cancer patients.

To validate GLUT1 and MCT4 protein expression in lung cancer tissues, we performed IHC analysis. In normal epithelial cells, GLUT1 expression was low, while red blood cells showed high GLUT1 expression, indicating different expression patterns across cell types (Supplementary Figure S3C). In lung cancer tissues, we observed both high and low GLUT1 expression, with GLUT1 also expressed in the tumor stroma, possibly related to tumor invasiveness and metastasis (Figure 4E). MCT4 was positively expressed on the tumor cell membrane, weakly in normal epithelium, and strongly in the surrounding microenvironment (Figure 4F). MCT4 may influence the TME’s acidity, affecting cellular metabolism and remodeling the TME. Finally, we demonstrated the coexpression of GLUT1 and MCT4 in LUAD tissues (Figure 4G). Immunofluorescence analysis revealed colocalization of GLUT1 and MCT4 on the lung cancer cell membrane, further supporting our hypothesis of their coexpression. These findings improve our understanding of GLUT1 and MCT4 expression patterns in NSCLC.

To explore the relationship between MCT4 and GLUT1 expression and clinical characteristics, we performed a chi-square analysis of 54 lung cancer patients (Table 1). The results showed that MCT4 and GLUT1 expression were not associated with clinical parameters or EGFR status (P > 0.05), possibly due to the small sample size.

**TABLE 1:** Relationships between MCT4 and GLUT1 protein expression and clinical parameters in lung cancer patients.

Parameter	MCT4			GLUT1		
	High	Low	*P*	High	Low	*P*
	(n = 26)	(n = 28)		(n = 33)	(n = 21)	
Age			0.098			0.713
<60	11	6		11	6	
≥60	15	22		22	15	
Gender			0.983			0.474
Male	14	15		19	10	
Female	12	13		14	11	
Smoking			0.439			0.653
Yes	11	9		13	7	
No	15	19		20	14	
Histology			0.134			0.250
Adenocarcinoma	24	28		31	21	
Squamous carcinoma	2	0		2	0	
EGFR status			0.439			0.653
Wild type	11	9		13	7	
Mutant type	15	19		20	14	

We extracted lung cancer tissues from mice for further validation. The mice were divided into PBS and 1 μM indacaterol groups, and IHC was performed on the tissues following drug treatment. IHC analysis revealed GLUT1 and MCT4 expression patterns in tumor tissues (Figure 4H). Indacaterol likely influenced these patterns, showing higher GLUT1 and lower MCT4 expression. The quantitative analysis of immunofluorescence data showed similar results (Figure 4I)

### 3.5 Indacaterol combined with a PD-L1 inhibitor suppresses lung cancer cells

PD-L1 is a receptor protein expressed on tumor cells that interacts with PD-1 on immune cells, facilitating tumor immune evasion (Tang et al., 2022). According to bioinformatics analysis linking *SLC2A1* to the TME, we hypothesized that indacaterol may interfere with immune evasion. Therefore, we further explored the effects of combining indacaterol with a PD-L1 inhibitor.

**FIGURE 5:**
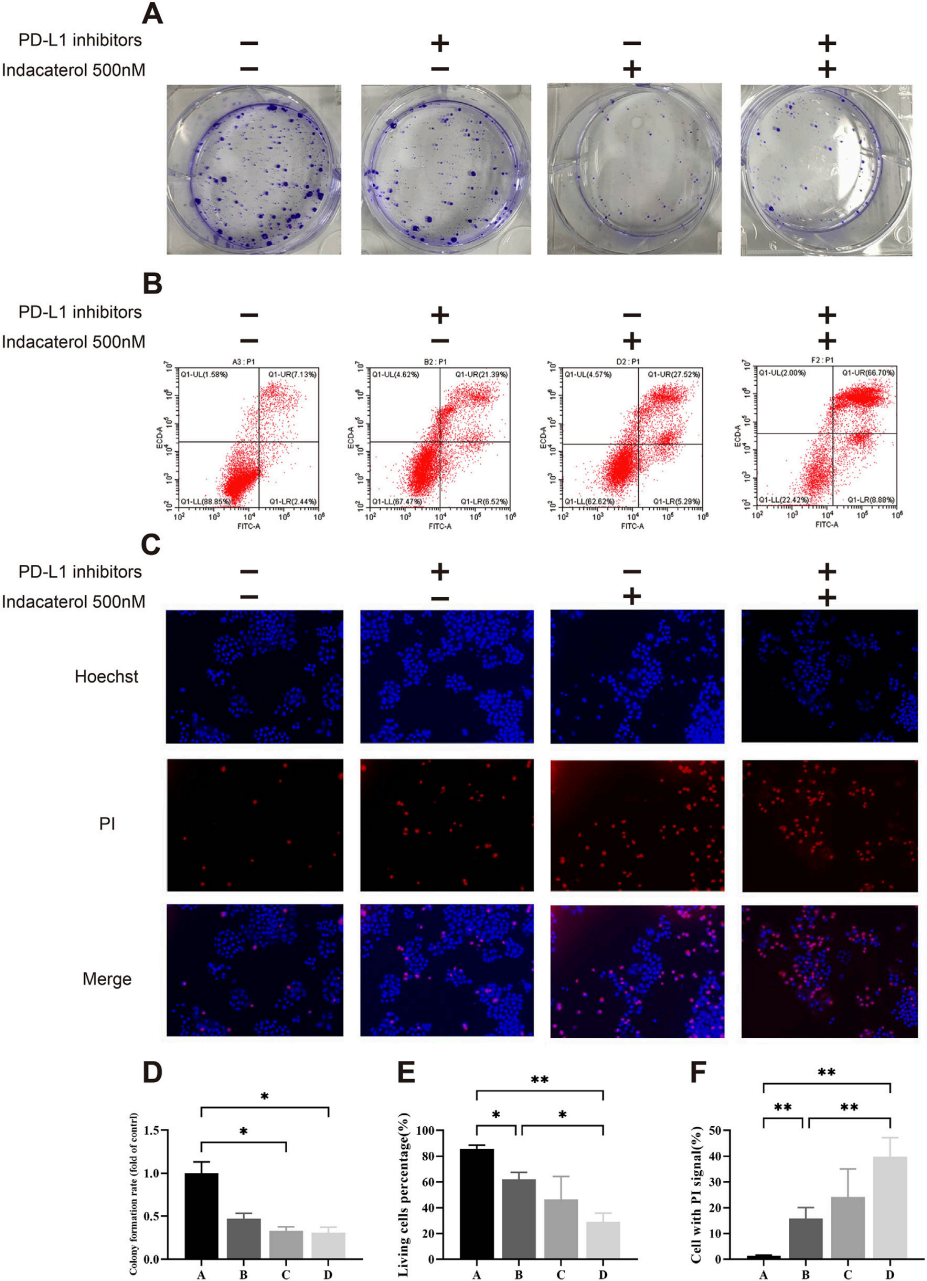
*In vitro* validation of the combined effects of indacaterol and PD-L1 inhibitors. **(A, D)** Colony formation assay showing the effects of indacaterol alone and in combination on colony formation in H460 cells. **(B, E)** Flow cytometry analysis showing the effects of indacaterol, a PD-L1 inhibitor, and their combination on H460 cell apoptosis. **(C, F)** Immunofluorescence image showing the effects of indacaterol, a PD-L1 inhibitor, and their combination on necrosis in H460 cells. (*p < 0.05, **p < 0.01) (In Fig D, E, and F, “A” means control, “B” means PD-L1 inhibitor, “C” means Indacaterol, and “D” means combination).

Using the H460 lung cancer cell line, we conducted a colony formation assay to assess the effects of the PD-L1 inhibitor and indacaterol, both alone and in combination, on clonogenic potential. Indacaterol can inhibit colony formation (Figure 5A, D). Flow cytometry showed a marked increase in apoptotic cells (p < 0.05), with greater apoptosis in the combination group than in the PD-L1 inhibitor alone group (p < 0.05) (Figure 5B, E). Fluorescence staining confirmed that both treatments promoted necrosis (p < 0.01), with the combination therapy having the most pronounced effect (p < 0.01) (Figure 5C, F). Transwell assay showed that the combination decreases cell invasiveness (p < 0.001) (Supplementary Figures S4A, B).

In summary, indacaterol alone can inhibit the proliferation of lung cancer cells. And the combination of indacaterol and a PD-L1 inhibitor promoted apoptosis and necrosis, and reduced cell invasiveness. These findings suggest that combining indacaterol with immune checkpoint inhibitors may offer a novel therapeutic strategy for lung cancer with promising clinical implications.

### 3.6 Indacaterol inhibits tumor growth in mice

**FIGURE 6:**
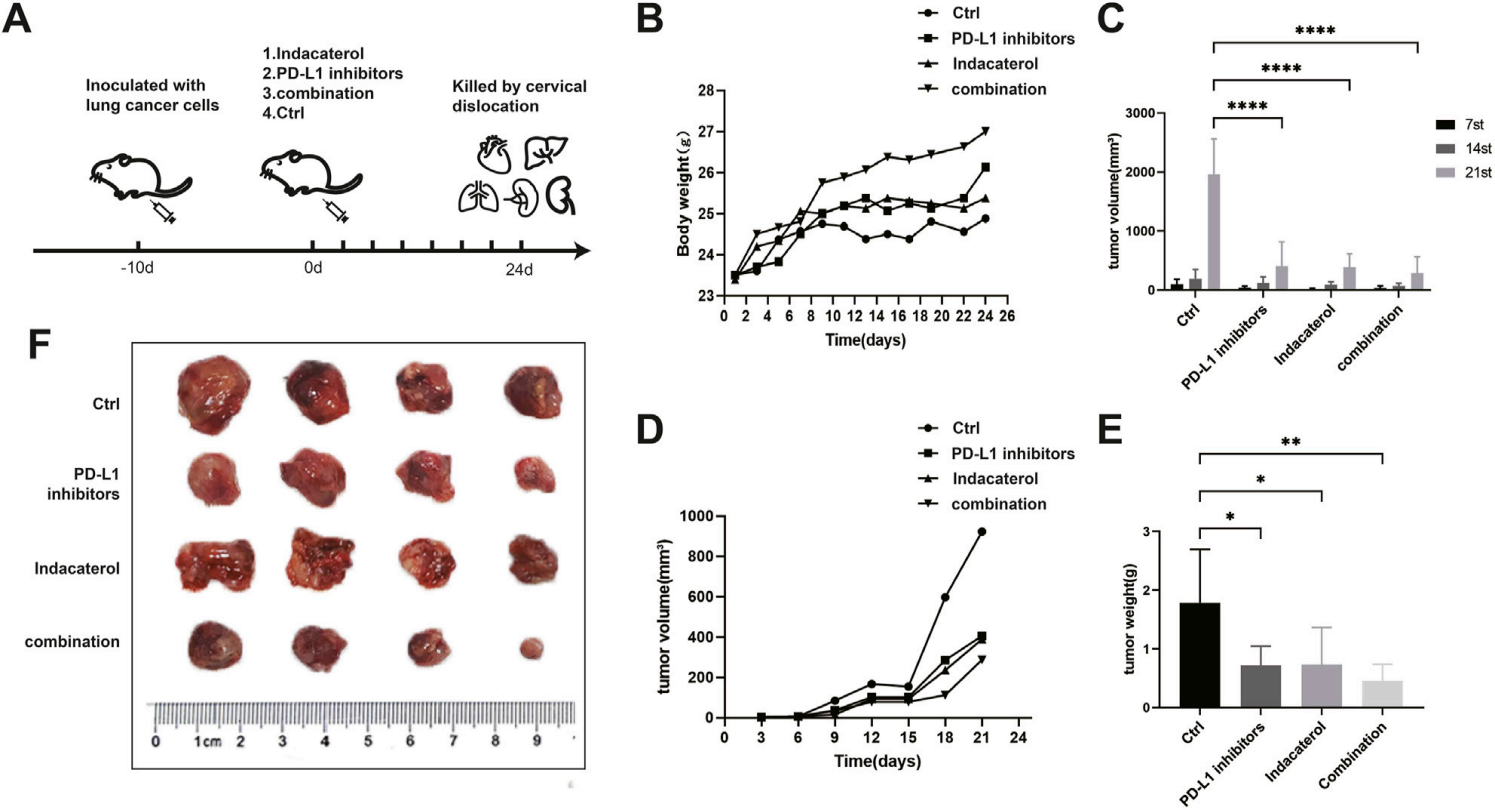
*In vivo* validation of the combined effects of indacaterol and PD-L1 inhibitors. **(A)** Schematic representation of mouse model construction, dividing the mice into Indacaterol, PD-L1 inhibitor, combination, and control groups. **(B)** Weight tracking of the mice during the treatment period. **(C, D)** Tumor volume measurements of the mice during treatment were performed via calipers, and the longest and shortest diameters were calculated. **(E, F)** Tumor weight and volume measurements postmortem. (*p < 0.05, **p < 0.01, ***p < 0.005, ****p < 0.001).

To investigate the impact of indacaterol on tumor growth *in vivo*, we constructed a Lewis lung cancer mouse model and divided the mice into four groups: control, indacaterol, PD-L1 inhibitor, and combination treatment (Figure 6A). Throughout the treatment, mice in the treatment groups gained more weight than those in the control group, with the combination group showing the greatest increase (Figure 6B). Tumor volume measurements revealed a significant reduction in the combination group (Figure 6C, D). At the end of the experiment, tumor weight was significantly lower in all treatment groups, with the combination group showing the largest decrease (Figure 6E, F).

HE staining of tumor tissues revealed extensive necrosis in the indacaterol and combination treatment groups, confirming the antitumor activity of indacaterol (Supplementary Figure S4C). Compared to the control group, the combination group showed lower serum ALT and AST levels (Supplementary Figures S4D, E) and a significant increase in direct bilirubin levels (p < 0.01) (Supplementary Figure S4F). Additionally, IL-2 and IFN-γ levels were elevated (p < 0.05) in the combination group (Supplementary Figures S4G, H). These results indicate that the anti-cancer effect of indacaterol is comparable to that of PD-L1 inhibitors. Moreover, the combination of the two may enhance the anti-cancer effect, but the difference is not significant, which might be related to the sample size.

Dissection and weighing of organs (heart, liver, spleen, lung, and kidney) showed reduced liver and spleen volumes after treatment. Organ index calculations revealed significantly lower liver and spleen indices in the combination treatment group compared to the control, indicating that the treatment affected growth and metabolism (Supplementary Figures S5A, B).

## 4 Discussion

In this study, we explored the potential of the long-acting β2-adrenergic receptor agonist indacaterol as an anticancer agent for NSCLC. Our findings elucidate the mechanisms of its antitumor effects and highlight the advantages of combining it with PD-L1 inhibitors.

Early clinical studies suggest that COPD patients receiving indacaterol have a lower incidence of lung cancer. However, a search of the pathology database at Zhongnan Hospital, Wuhan University, for cases of “NSCLC,” “COPD,” and “Indacaterol” yielded no relevant cases, raising the possibility of a link between indacaterol and lung cancer.

In preliminary experiments, indacaterol significantly inhibited lung cancer cell viability, migration, proliferation, and invasion in H1299, A549, and H460 cell lines. Online docking simulations identified potential targets of indacaterol, including HIF, amyloid β-peptide binding protein, GLUT, and G protein-coupled receptor 55. Further investigation of glucose transporters provided insights into the anticancer mechanism of indacaterol.

Analysis of the TCGA database revealed that GLUT1 expression was significantly higher in lung cancer tissues compared to normal tissues, and this overexpression was linked to TME remodeling and poor patient outcomes. GLUT1 overexpression in tumor cells enhances glucose uptake, supporting increased metabolic demands under hypoxic conditions (Ancey et al., 2018). Typically, during tumor cell death, many membrane proteins exhibit structural alterations or decreased expression. GLUT1, a representative marker, usually shows reduced expression during cell death (Li et al., 2023b). However, our study revealed an atypical increase in GLUT1 expression following indacaterol treatment, warranting further investigation. Molecular docking simulations predicted a stable hydrogen bond interaction between indacaterol and GLUT1, suggesting that indacaterol may affect GLUT1’s biological functions. To test this hypothesis, we conducted a CETSA, which showed a significant shift in the melting curve of GLUT1 in the indacaterol-treated group, indicating binding between indacaterol and GLUT1. Fluorescence experiments further supported this.

We speculate that indacaterol may exert antitumor effects through the potential targeting of GLUT1. By binding to GLUT1, indacaterol could disrupt metabolic processes essential for cancer cell survival and proliferation. The observed upregulation of GLUT1 after treatment might represent a compensatory feedback response triggered by pharmacological intervention. Preliminary molecular modeling and CETSA data are consistent with a putative interaction between indacaterol and GLUT1. Paradoxically, the upregulation of GLUT1 — a pro-tumorigenic protein—may indicate functional downregulation, potentially due to compensatory feedback mechanisms or binding-induced inactivation. However, further experiments related to glucose uptake or glycolytic metabolism are needed for verification.

*In vitro* and *in vivo* experiments confirmed that indacaterol treatment upregulated GLUT1, downregulated MCT4, reduced cell viability, and increased cell death, consistent with our initial predictions. TCGA database analysis and IHC experiments on LUAD tissues showed coexpression of GLUT1 and MCT4, suggesting joint regulation of lung cancer metabolism. Indacaterol affects the metabolism of lung cancer cells, while MCT4 downregulation promoted cell death. This downregulation blocked lactate export, leading to intracellular lactate accumulation, which increased acidity and hindered cancer cell survival and proliferation. Acidic metabolites in the TME impair immune cell function, disrupting their metabolism and differentiation (Cheng et al., 2023). Indacaterol’s effect on the acidic environment may alter immune cell metabolism and function, influencing the tumor’s immune response.

A key finding of this study is the synergistic effect between indacaterol and PD-L1 inhibitors. PD-L1 is an immune checkpoint protein exploited by cancer cells to evade immune detection. The combination of indacaterol and PD-L1 inhibitors significantly inhibited lung cancer activity, with effects greater than either treatment alone. In the *in vivo* experiments of lung cancer mouse models, it was confirmed that indacaterol had comparable anti-cancer effects with PD-L1 inhibitors. The tumor size and weight were significantly reduced, and the overall health condition was also improved, manifested as weight gain and reduced tumor invasiveness. The combined treatment of the two could enhance the anti-cancer effect of PD-L1 inhibitors, but the difference was not significant. This might be due to the sample size issue. Further experiments are needed for verification in the future.

We speculate that this dual strategy targeting metabolic pathways and enhancing immune responses makes indacaterol promising as one of the future therapeutic options for lung cancer. Indacaterol’s well-established safety in COPD patients supports its potential for cancer therapy. However, further clinical trials are needed to validate these preclinical findings and optimize dosing regimens. Our research also opens the door to combining indacaterol with other metabolic inhibitors or immune checkpoint inhibitors. Understanding how indacaterol interacts with GLUT1 and other metabolic proteins could help develop more effective cancer therapies.

## 5 Conclusion

In conclusion, our study identified the therapeutic potential of indacaterol as a novel anticancer drug, particularly for lung cancer treatment. When used in combination with PD-L1 inhibitors, indacaterol has significantly greater efficacy than monotherapy. This combination therapy leverages both metabolic inhibition and immune activation, thereby presenting a new model for lung cancer treatment (Supplementary Figure S6).

## Data Availability

The original contributions presented in the study are included in the article/Supplementary Material, further inquiries can be directed to the corresponding author/s.

## Abbreviations

NSCLC, Non-small cell lung cancer; COPD, Chronic obstructive pulmonary disease; TME, Tumor microenvironment; CAFs, Cancer-associated fibroblasts; OS, Overall survival; DSS, Disease-specific survival; DFI, Disease-free interval; PFI, Progression-free interval; GSEA, Gene set enrichment analysis; GSVA, Gene set variation analysis; T, Tumor size; N, Lymph node; M, Metastasis; CETSA, Cellular thermal shift assay; IHC, Immunohistochemistry; LUAD, Lung adenocarcinoma.

## References

[B1] Alam S. Doherty E. Ortega-Prieto P. Arizanova J. Fets L. (2023). Membrane transporters in cell physiology, cancer metabolism and drug response. Dis. Model Mech. 16 (11), dmm050404. 10.1242/dmm.050404 38037877 PMC10695176

[B2] Ancey P. B. Contat C. Meylan E. (2018). Glucose transporters in cancer - from tumor cells to the tumor microenvironment. Febs J. 285 (16), 2926–2943. 10.1111/febs.14577 29893496

[B3] Ayama-Canden S. Tondo R. Pineros Leyton M. L. Ninane N. Demazy C. Dieu M. (2023). Indacaterol inhibits collective cell migration and IGDQ-mediated single cell migration in metastatic breast cancer MDA-MB-231 cells. Cell Commun. Signal 21 (1), 301. 10.1186/s12964-023-01340-9 37904233 PMC10614342

[B4] Babl N. Decking S. M. Voll F. Althammer M. Sala-Hojman A. Ferretti R. (2023). MCT4 blockade increases the efficacy of immune checkpoint blockade. J. Immunother. Cancer 11 (10), e007349. 10.1136/jitc-2023-007349 37880183 PMC10603342

[B5] Baczewska M. Supruniuk E. Bojczuk K. Guzik P. Milewska P. Konończuk K. (2022). Energy substrate transporters in high-grade ovarian cancer: gene expression and clinical implications. Int. J. Mol. Sci. 23 (16), 8968. 10.3390/ijms23168968 36012230 PMC9408757

[B6] Bray F. Laversanne M. Sung H. Ferlay J. Siegel R. L. Soerjomataram I. (2024). Global cancer statistics 2022: GLOBOCAN estimates of incidence and mortality worldwide for 36 cancers in 185 countries. CA Cancer J. Clin. 74 (3), 229–263. 10.3322/caac.21834 38572751

[B7] Cao S. Chen Y. Ren Y. Feng Y. Long S. (2021). GLUT1 biological function and inhibition: research advances. Future Med. Chem. 13 (14), 1227–1243. 10.4155/fmc-2021-0071 34018847

[B8] Cheng H. Qiu Y. Xu Y. Chen L. Ma K. Tao M. (2023). Extracellular acidosis restricts one-carbon metabolism and preserves T cell stemness. Nat. Metab. 5 (2), 314–330. 10.1038/s42255-022-00730-6 36717749 PMC9970874

[B9] Cong D. Zhao Y. Zhang W. Li J. Bai Y. (2023). Applying machine learning algorithms to develop a survival prediction model for lung adenocarcinoma based on genes related to fatty acid metabolism. Front. Pharmacol. 14, 1260742. 10.3389/fphar.2023.1260742 37920207 PMC10619909

[B10] Fontana F. Giannitti G. Marchesi S. Limonta P. (2024). The PI3K/Akt pathway and glucose metabolism: a dangerous liaison in cancer. Int. J. Biol. Sci. 20 (8), 3113–3125. 10.7150/ijbs.89942 38904014 PMC11186371

[B11] Forder A. Zhuang R. Souza V. G. P. Brockley L. J. Pewarchuk M. E. Telkar N. (2023). Liquid biopsy in lung cancer: biomarkers for the management of recurrence and metastasis. Int. J. Mol. Sci. 24 (3), 8894. 10.3390/ijms24108894 37240238 PMC10219023

[B12] Gargalionis A. N. Papavassiliou K. A. Basdra E. K. Papavassiliou A. G. (2024). Advances in non-small cell lung cancer mechanomedicine: deciphering the signaling networks that govern tumor-TME interactions. J. Exp. and Clin. cancer Res. CR 43 (1), 316. 10.1186/s13046-024-03242-1 39616383 PMC11608457

[B13] Granja S. C. Longatto-Filho A. de Campos P. B. Oliveira C. P. Stefano J. T. Martins-Filho S. N. (2022). Non-alcoholic fatty liver disease-related hepatocellular carcinoma: immunohistochemical assessment of markers of cancer cell metabolism. Pathobiology 89 (3), 157–165. 10.1159/000521034 35042213

[B14] Kim J. Y. Shin J. H. Kim M. J. Kang Y. Lee J. S. Son J. (2023). β-arrestin 2 negatively regulates lung cancer progression by inhibiting the TRAF6 signaling axis for NF-κB activation and autophagy induced by TLR3 and TLR4. Cell death and Dis. 14 (7), 422. 10.1038/s41419-023-05945-3 PMC10344878 37443143

[B15] Leone R. D. Powell J. D. (2020). Metabolism of immune cells in cancer. Nat. Rev. Cancer 20 (9), 516–531. 10.1038/s41568-020-0273-y 32632251 PMC8041116

[B16] Li H. Tong C. W. Leung Y. Wong M. H. To K. K. Leung K. S. (2017). Identification of clinically approved drugs indacaterol and canagliflozin for repurposing to treat epidermal growth factor tyrosine kinase inhibitor-resistant lung cancer. Front. Oncol. 7, 288. 10.3389/fonc.2017.00288 29238696 PMC5712561

[B17] Li Y. Tang S. Shi X. Lv J. Wu X. Zhang Y. (2023b). Metabolic classification suggests the GLUT1/ALDOB/G6PD axis as a therapeutic target in chemotherapy-resistant pancreatic cancer. Cell Rep. Med. 4 (9), 101162. 10.1016/j.xcrm.2023.101162 37597521 PMC10518604

[B18] Li Y. Yan B. He S. (2023a). Advances and challenges in the treatment of lung cancer. Biomed. Pharmacother. 169, 115891. 10.1016/j.biopha.2023.115891 37979378

[B19] Madunić I. V. Madunić J. Breljak D. Karaica D. Sabolić I. (2018). Sodium-glucose cotransporters: new targets of cancer therapy? Arh. Hig. Rada Toksikol. 69 (4), 278–285. 10.2478/aiht-2018-69-3204 30864374

[B20] Pirker R. (2020). Chemotherapy remains a cornerstone in the treatment of nonsmall cell lung cancer. Curr. Opin. Oncol. 32 (1), 63–67. 10.1097/CCO.0000000000000592 31599771

[B21] Revathidevi S. Munirajan A. K. (2019). Akt in cancer: mediator and more. Semin. Cancer Biol. 59, 80–91. 10.1016/j.semcancer.2019.06.002 31173856

[B22] Singh D. Wild J. M. Saralaya D. Lawson R. Marshall H. Goldin J. (2022). Effect of indacaterol/glycopyrronium on ventilation and perfusion in COPD: a randomized trial. Respir. Res. 23 (1), 26. 10.1186/s12931-022-01949-3 35144620 PMC8832861

[B23] Singh M. Afonso J. Sharma D. Gupta R. Kumar V. Rani R. (2023). Targeting monocarboxylate transporters (MCTs) in cancer: how close are we to the clinics? Semin. Cancer Biol. 90, 1–14. 10.1016/j.semcancer.2023.01.007 36706846

[B24] Tang Q. Chen Y. Li X. Long S. Shi Y. Yu Y. (2022). The role of PD-1/PD-L1 and application of immune-checkpoint inhibitors in human cancers. Front. Immunol. 13, 964442. 10.3389/fimmu.2022.964442 36177034 PMC9513184

[B25] Wan L. Yu W. Shen E. Sun W. Liu Y. Kong J. (2019). SRSF6-regulated alternative splicing that promotes tumour progression offers a therapy target for colorectal cancer. Gut 68 (1), 118–129. 10.1136/gutjnl-2017-314983 29114070

[B26] Xiao Y. Yu D. (2021). Tumor microenvironment as a therapeutic target in cancer. Pharmacol. Ther. 221, 107753. 10.1016/j.pharmthera.2020.107753 33259885 PMC8084948

[B27] Zouein J. Haddad F. G. Eid R. Kourie H. R. (2022). The combination of immune checkpoint inhibitors and chemotherapy in advanced non-small-cell lung cancer: the rational choice. Immunotherapy 14 (2), 155–167. 10.2217/imt-2021-0014 34865502

