# Supplementary material for: From COPD to cancer: indacaterol’s unexpected role in combating NSCLC
Source: Front Pharmacol. 2025 Apr 3;16:1579126. doi: 10.3389/fphar.2025.1579126 (PMC12018804; doi:10.3389/fphar.2025.1579126)
Supplement: Supplementary file 1 [file DataSheet1.docx]

Supplementary Material

# Supplementary Figures


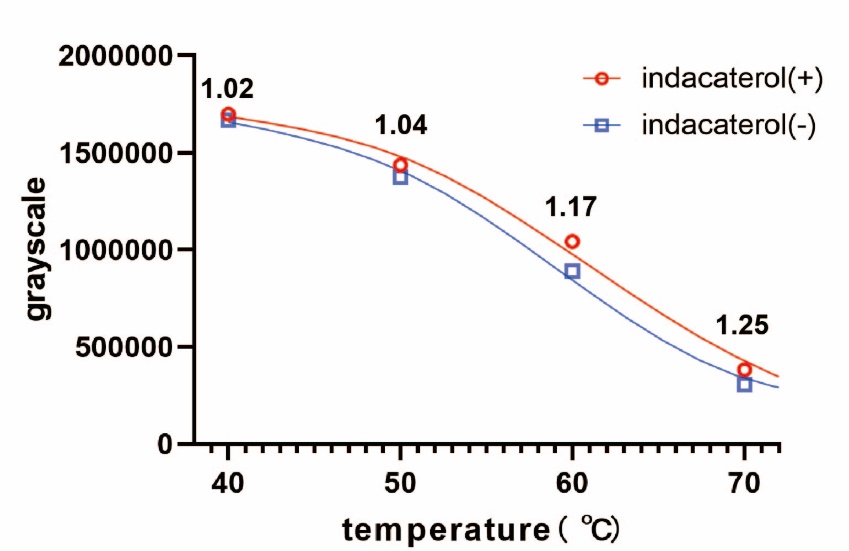


**Supplementary Figure 1.** The CESTA results underwent grayscale validation, with numerical annotations on the curve denoting relative values.

**
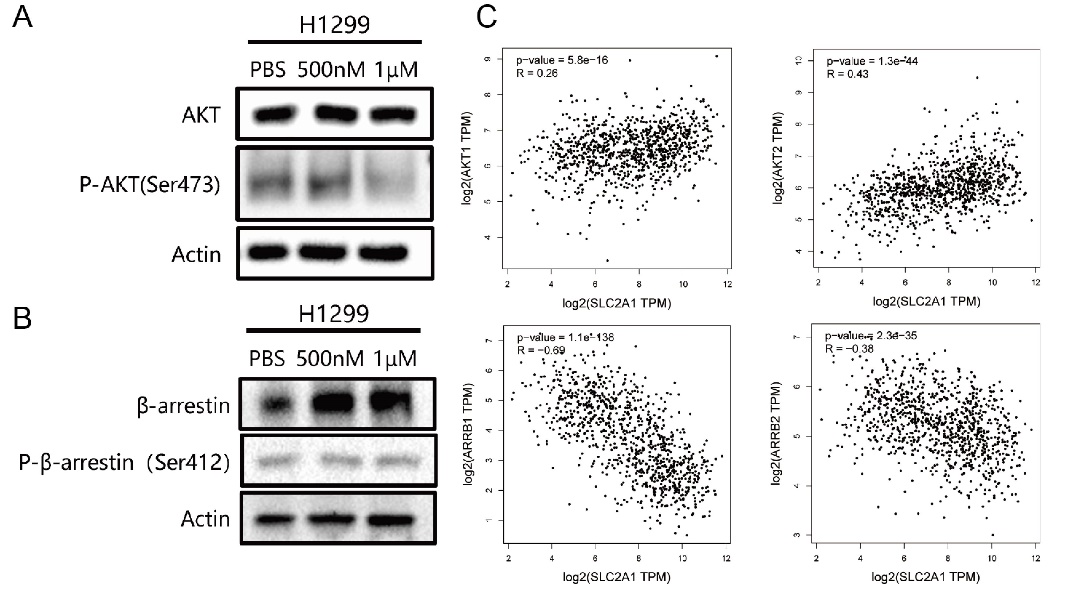
**

**Supplementary Figure 2.** Detection of metabolism-related proteins after indacaterol treatment in H1299 cells. **(A)** Western blot of the AKT and P-AKT proteins in H1299 cells treated with Indacaterol. **(B)** Western blot of β-arrestin and P-β-arrestin proteins in H1299 cells treated with indacaterol. **(C)** Correlation analysis between *SLC2A1* and *AKT1*, *AKT2*, *ARRB1*, and *ARRB2* in LUAD samples from the TCGA.


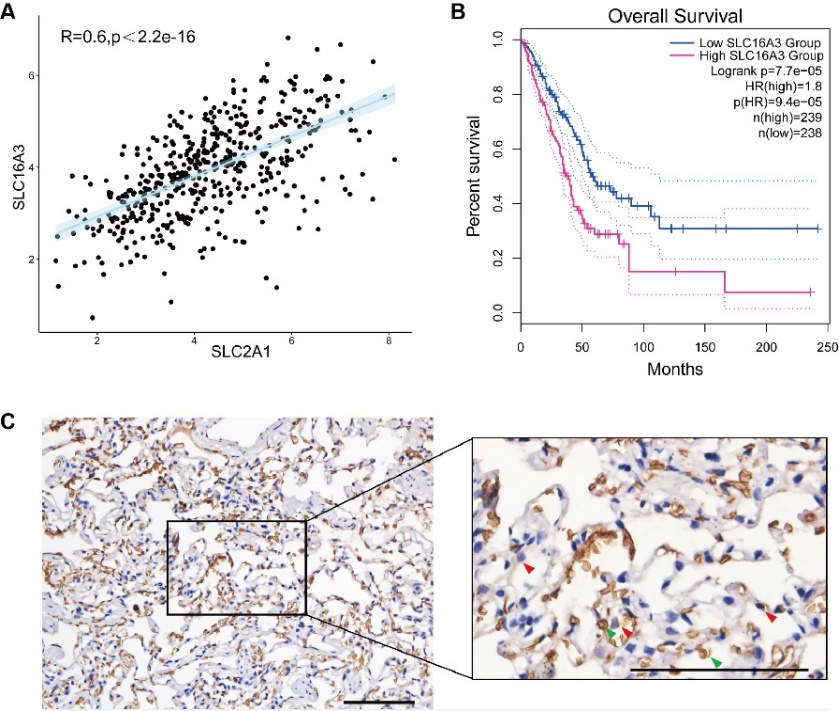


**Supplementary Figure 3.** Bioinformatics and histological analyses of *SLC2A1* and *SLC16A3* in lung cancer. **(A)** Correlation between *SLC2A1* and *SLC16A3* in LUAD. **(B)** Kaplan‒Meier curves for the high and low-expression groups of *SLC16A3* in LUAD. **(C)** Immunohistochemistry of GLUT1 protein in normal human lung tissue.


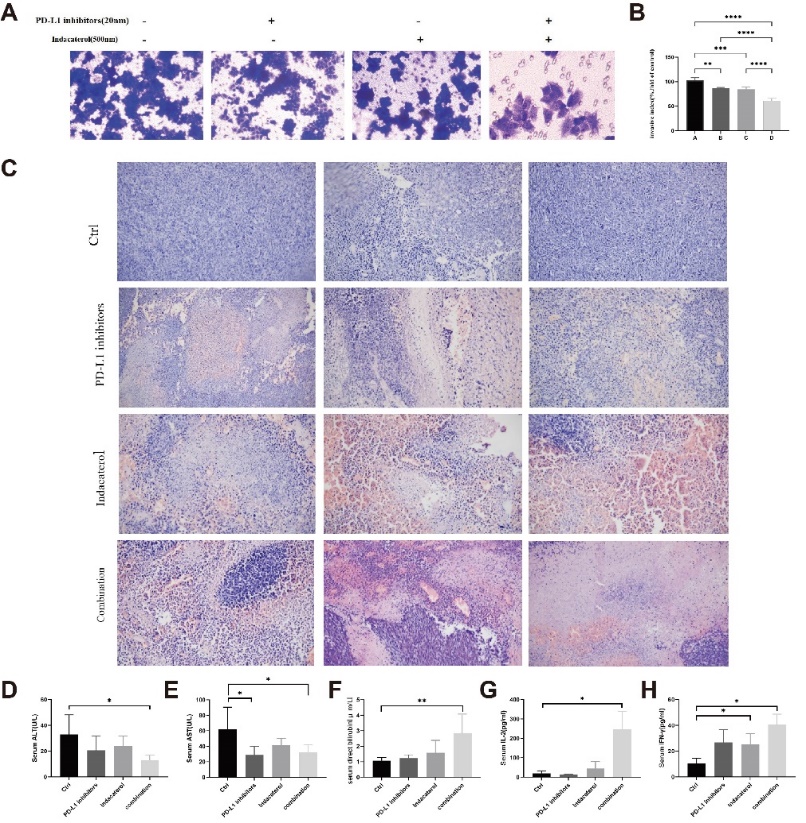


**Supplementary Figure 4.** Transwell assay and tissue staining in combination with PD-L1 inhibitor treatment. **(A)** Transwell assay showing the effects of indacaterol, PD-L1 inhibitors, and their combination on the invasive ability of H460 cells. **(B)** Quantitative analysis of the Transwell assay results. **(C)** HE staining of tumor tissues from mice treated with indacaterol, PD-L1 inhibitors, or their combination. ELISA analysis of serum changes in tumor-bearing mice, showing the levels of ALT **(D)**, **AST** **(E)**, direct bilirubin **(F)**, IL-2 **(G)**, and IFN-γ **(H).**

**
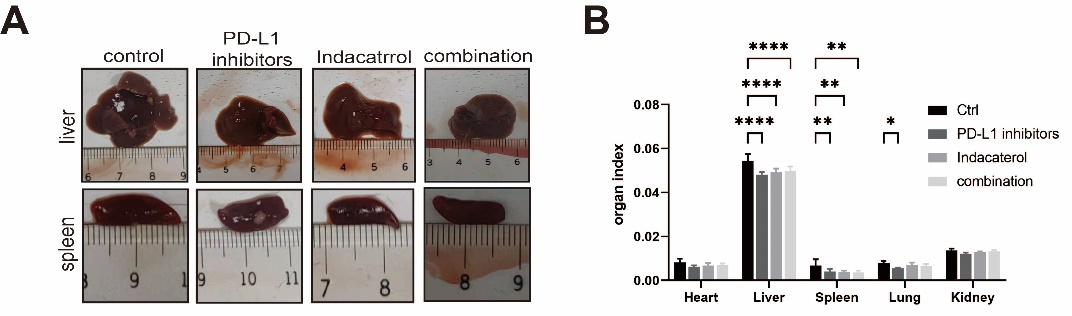
**

**Supplementary Figure 5.** Organ index in combination with PD-L1 inhibitor treatment. **(A-B)** Organ index calculations for the heart, liver, spleen, lungs, and kidneys of treated mice.


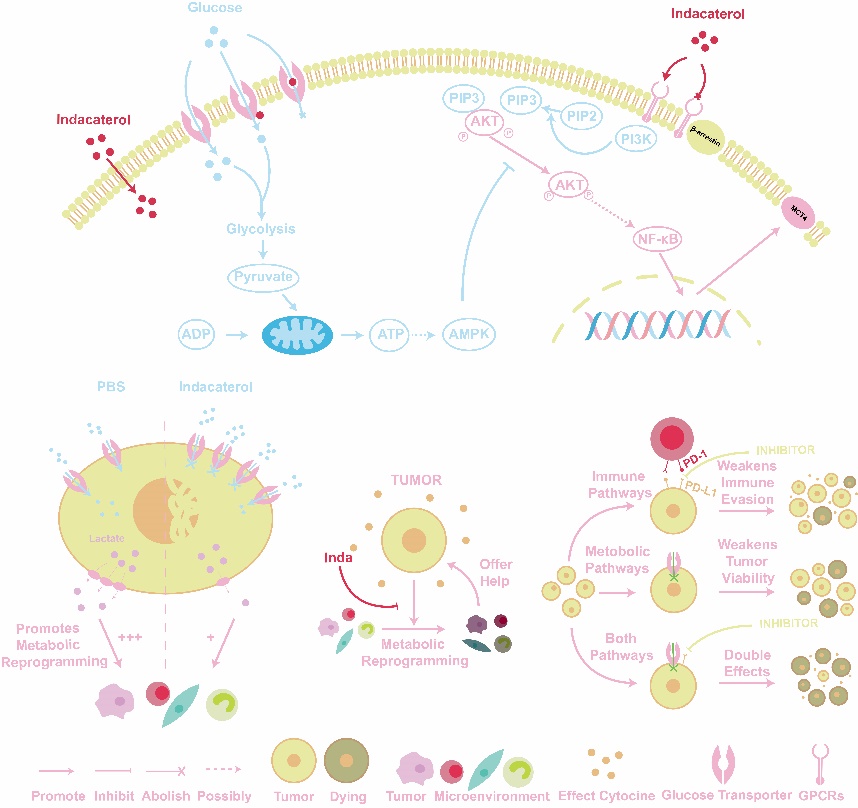


**Supplementary Figure 6.** Indacaterol as a Novel Lung Cancer Therapy: Potential Mechanisms and Synergistic Potential with PD-L1 Inhibitors. Indacaterol upregulates GLUT1 expression and inhibits AKT phosphorylation, affecting the PI3K/AKT pathway. Additionally, it suppresses MCT4 expression, potentially altering the tumor microenvironment. Combining indacaterol with a PD-L1 inhibitor enhances anti-tumor effects, promoting apoptosis and reducing invasiveness. These findings suggest that indacaterol, alone or with immune checkpoint inhibitors, may offer a promising lung cancer therapy
